# Supplementary material for: The genome sequence of the yellow mealworm beetle, Tenebrio molitor Linnaeus, 1758
Source: Wellcome Open Res. 2024 Aug 12;9:459. [Version 1] doi: 10.12688/wellcomeopenres.22863.1 (PMC11558167; doi:10.12688/wellcomeopenres.22863.1)
Supplement: Peer review history

# Reviewer report: Reviewer response for version 1

**DOI:** 10.21956/wellcomeopenres.25176.r99952

**Published:** 2024-11-12

**Authors:** Gengyun Niu

**Affiliations:** 1 Jiangxi Normal University, Nanchang, Jiangxi, China

This manuscript provides a high-quality genome sequence of the yellow mealworm, making an important contribution to the fields of insect genomics and evolutionary biology. Through well-designed experiments and advanced data analysis pipelines, the authors have successfully assembled the chromosome-level genome of the yellow mealworm and evaluated it in detail.

 However, perhaps due to stylistic constraints, the paper also suffers from some shortcomings, such as the lack of genome annotation, which limits a comprehensive understanding of its structure and function.

 Nevertheless, I recommend acceptance of this paper and encourage the authors to continue their in-depth research in this area.

Are sufficient details of methods and materials provided to allow replication by others?

Yes

Is the rationale for creating the dataset(s) clearly described?

Yes

Are the datasets clearly presented in a useable and accessible format?

Yes

Are the protocols appropriate and is the work technically sound?

Yes

Reviewer Expertise:

mitochondrial genomics; Insect Taxonomy; Phylogeny

I confirm that I have read this submission and believe that I have an appropriate level of expertise to confirm that it is of an acceptable scientific standard.

# Reviewer report: Reviewer response for version 1

**DOI:** 10.21956/wellcomeopenres.25176.r99938

**Published:** 2024-09-24

**Authors:** Romain Villoutreix

**Affiliations:** 1 Universite de Montpellier, Montpellier, Occitanie, France

Mann and colleagues present a genome assembly for the yellow mealworm beetle *Tenebrio molitor.*

 Great care was taken in identifying an individual of the target species, with comparison of a COI marker from previously published data. 

 Great care was also taken in producing and curating this assembly, with special care taken to remove possible contamination. The authors used cutting edge data and pipelines to produce an excellent chromosomal level assembly.

 This resource will be of great use to the community. I therefore recommend the indexing of this data note.

Are sufficient details of methods and materials provided to allow replication by others?

Yes

Is the rationale for creating the dataset(s) clearly described?

Yes

Are the datasets clearly presented in a useable and accessible format?

Yes

Are the protocols appropriate and is the work technically sound?

Yes

Reviewer Expertise:

Evolutionary Genomics

I confirm that I have read this submission and believe that I have an appropriate level of expertise to confirm that it is of an acceptable scientific standard.
